# Ionic Liquid as an Efficient Medium for the Synthesis of Quinoline Derivatives via α-Chymotrypsin-Catalyzed Friedländer Condensation

**DOI:** 10.3390/molecules22050762

**Published:** 2017-05-08

**Authors:** Zhang-Gao Le, Meng Liang, Zhong-Sheng Chen, Sui-Hong Zhang, Zong-Bo Xie

**Affiliations:** 1 Jiangxi 2011 Joint Center for the Innovative Mass Spectrometry and Instrumentation, East China University of Technology, Nanchang 330013, China; zhgle@ecit.cn; 2 School of Chemistry, Biology and Material Science, East China University of Technology, Nanchang 330013, China; 13247718220@163.com (M.L.); zhshcheng@ecit.cn (Z.-S.C.); zsh18170400156@163.com (S.-H.Z.)

**Keywords:** Friedländer reaction, quinolines, α-chymotrypsin, ionic liquid, biocatalysis, promiscuity

## Abstract

An efficient, convenient, and eco-friendly biocatalytic approach was developed for the synthesis of quinoline derivatives via the α-chymotrypsin-catalyzed Friedländer reaction. Interestingly, α-chymotrypsin exhibited higher catalytic activity in an ionic liquid (IL) aqueous solution as compared to that observed in our previous relevant study, which was conducted using an organic solvent, and a series of substrates gave similar excellent yields at lower reaction temperature and under reduced enzyme-loading conditions.

## 1. Introduction

Quinoline derivatives are of great significance in medicinal chemistry [1] as they usually display a broad range of excellent pharmacological activities, including anticancer [2], antiviral [3], anti-microbial [4], antifungal [5], anti-inflammatory [6], and anti-platelet aggregation [7] activities, which render them important drug intermediates. In addition, quinoline derivatives are valuable synthons for the preparation of nano- and mesostructures with enhanced electronic and photonic properties [8,9]. Various methods were developed for the synthesis of this class of compounds, such as the Skraup synthesis [10], Doebner–Von Miller reaction [11], Combes synthesis [12], and the Friedländer method [13]. Among these, the Friedländer method, which involves a condensation reaction between a 2-aminoaryl ketone and a α-methylene ketone, is the most simple and straightforward. Generally, this process can be carried out in the presence of base or acid catalysts to yield quinoline derivatives. However, this method often suffers from poor selectivity, complicated procedures, or harsh conditions [14,15] and is not practically feasible. Over recent years, biocatalysis has attracted increasing interest in synthetic chemistry because it offers high efficiency and excellent selectivity and requires mild reaction conditions; thus, biocatalysis has been identified as an eco-friendly and sustainable alternative to traditional organic synthesis [16,17]. In particular, several valuable studies on enzymatic promiscuity have been reported; for instance, hydrolase catalyzes unconventional reactions such as the Aldol reaction [18,19], the Mannich reaction [20], and the Henry reaction [21]. In our previous work, we demonstrated the first α-chymotrypsin-catalyzed Friedländer condensation between a 2-aminoaryl ketone and an α-methylene ketone in an organic solvent [22], but the environmental problems related to the use of organic solvents are a matter of serious concern. Room-temperature ionic liquids (RTILs), superior alternatives to organic solvents, have many potential benefits for biochemical processes, especially where the reaction substrates and biocatalysts can be dissolved better, leading to a more efficient reaction process. Recently, some hydrolases have been reported to catalyze organic reactions for the synthesis of peptides [23] and esters [24,25] in an ionic liquid (IL) aqueous solution. The enzyme activity in this medium was clearly enhanced in comparison with that in organic solvents, and the excellent results obtained suggested that ILs hold great potential as efficient reaction media for biocatalysis.

**Scheme 1:**
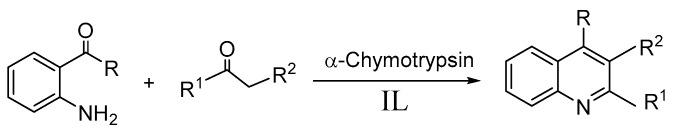
Friedländer condensation reaction between 2-aminoaryl ketones and α-methylene ketone in ionic liquid (IL) aqueous solution.

As part of our continuing interest in enzymatic synthesis and green synthetic methodologies, we report a mild and efficient biocatalytic route for the Friedländer reaction. Here, α-chymotrypsin is reported for the first time to catalyze the condensation reaction between a 2-aminoaryl ketone and a α-methylene ketone for the preparation of quinoline derivatives in an IL (1-ethyl-3-methylimidazolium tetrafluoroborate) aqueous solution ([EMIM][BF_4_]/H_2_O) (Scheme 1).

## 2. Results and Discussion

**Scheme 2:**
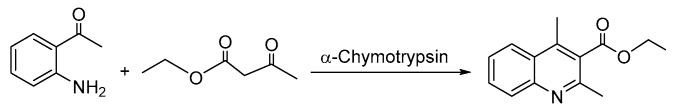
Model reaction between 2-aminoacetophenone and ethyl acetoacetate.

**Figure 1:**
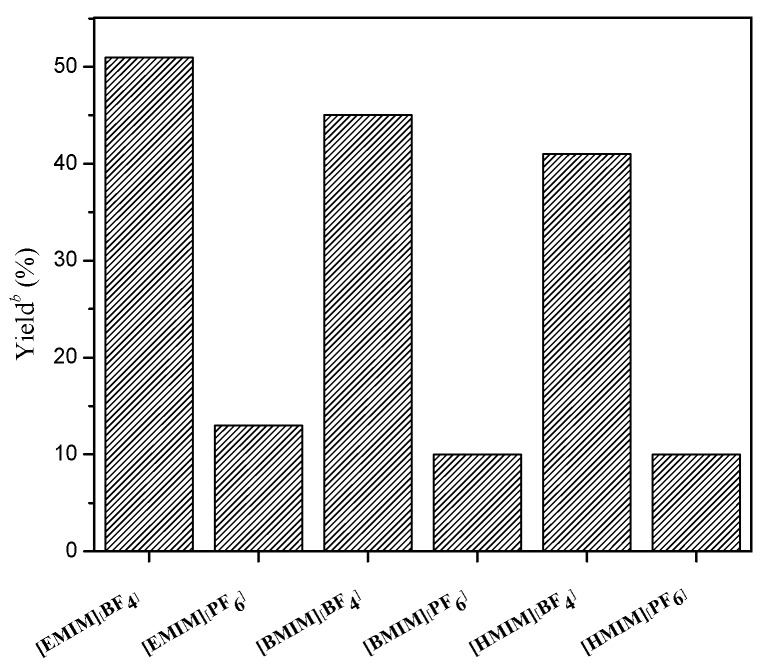
Reaction yields in different IL aqueous solutions ^a^. ^a^ *Reaction conditions*: 2-aminoacetophenone (0.3 mmol), ethyl acetoacetate (0.36 mmol), and α-chymotrypsin (10 mg) in 50% (H_2_O/(IL + H_2_O), *v*/*v*) IL aqueous solution at 60 °C for 24 h. ^b^ Isolated yield after column chromatography.

The reaction of 2-aminoacetophenone and ethyl acetoacetate was chosen as the model reaction for determining the optimal conditions (Scheme 2). It is well known that enzyme activity is strongly affected by the reaction medium. In order to select the optimal reaction medium, the initial experiment was carried out in six different dry ILs, including three tetrafluoroborate ILs (1-ethyl-3-methylimidazolium tetrafluoroborate, 1-butyl-3-methylimidazolium tetrafluoroborate, and 1-hexyl-3-methylimidazolium tetrafluoroborate) and three hexafluorophosphate ILs (1-ethyl-3-methylimidazolium hexafluorophosphate, 1-butyl-3-methylimidazolium hexafluorophosphate, and 1-hexyl-3-methylimidazolium hexafluorophosphate). Unfortunately, α-chymotrypsin exhibited extremely poor catalytic activity, and the product could not be detected by TLC. This was possibly due to the high viscosity of the reaction medium, which limited mass transfer between the substrates and the active site of the enzyme. Then, 10% (H_2_O/(IL + H_2_O), *v*/*v*) water was added to the ILs to reduce their viscosity, and the activity of α-chymotrypsin was found to be enhanced. To clearly distinguish between the various ILs, more water was added to each IL; better results were obtained when 50% water was used (Figure 1), and the best yield reached 51%. At the same time, we found that different ILs have a different effect on the enzyme activity, and the ILs with [BF_4_]^−^ and a cation containing short 1-alkyl groups could provide better results, among which 1-ethyl-3-methylimidazolium tetrafluoroborate contributed to superior enzyme activity. The probable reason for this is that the IL with the cation containing a shorter alkyl chain has higher polarity, thus increasing the solubility of the substrates and enzyme, and eventually accelerating the reaction process. As indicated above, we chose (1-ethyl-3-methylimidazolium tetrafluoroborate) [EMIM][BF_4_] as the optimal IL for further reactions.

**Figure 2:**
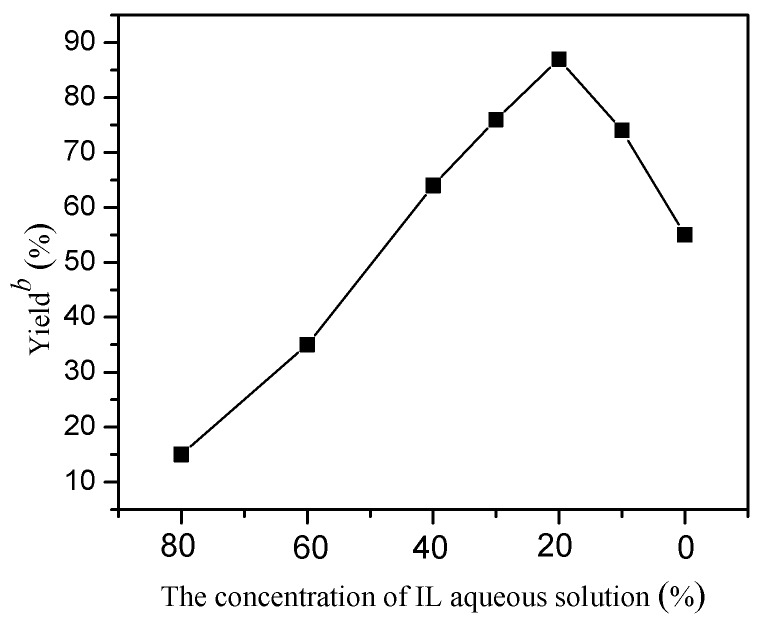
Reaction yield in IL aqueous solutions with different concentrations ^a^. ^a^ *Reaction conditions*: 2-aminoacetophenone (0.3 mmol), ethyl acetoacetate (0.36 mmol), and α-chymotrypsin (10 mg) in IL aqueous solutions of different concentrations (80 to 0%, [EMIM][BF_4_]/(H_2_O + [EMIM][BF_4_]), *v*/*v*) at 60 °C for 24 h. ^b^ Isolated yield after column chromatography.

To explain the low yield observed in some cases, the effect of water content was investigated in further detail (Figure 2). The water content was found to have a notable influence on the activity of α-chymotrypsin. An increase in the water content led to an enhancement in the synthetic activity of α-chymotrypsin. When the water content reached 80% (H_2_O/([BMIM][BF_4_] + H_2_O), *v*/*v*), α-chymotrypsin exhibited the best catalytic activity with an excellent yield of 87%; in other words, a 20% IL aqueous solution can give results that are superior to those observed previously when using an organic solvent. From the discussion above, we can conclude that the IL with optimal water content can provide an appropriate environment for the mass transfer of substrates to the active site of the enzyme, in which the enzyme activity was improved to some degree, thus allowing for the efficient synthesis of quinoline derivatives. Thus, the 20% IL aqueous solution was chosen as the optimum medium for the α-chymotrypsin-catalyzed Friedländer condensation.

**Figure 3:**
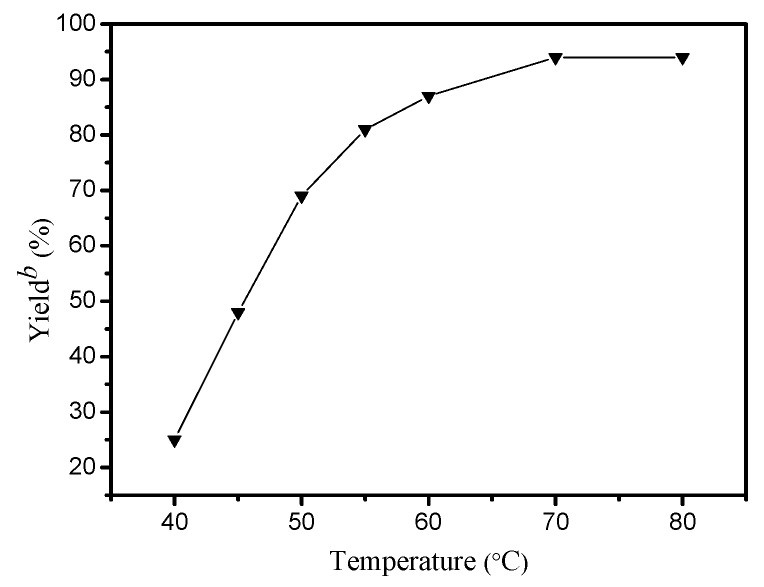
Reaction yields at different temperatures ^a^. ^a^ *Reaction conditions*: 2-aminoacetophenone (0.3 mmol), ethyl acetoacetate (0.36 mmol), and α-chymotrypsin (10 mg) in 20% ([EMIM][BF_4_]/(H_2_O + [EMIM][BF_4_]), *v*/*v*) IL aqueous solution at different temperatures for 24 h. ^b^ Isolated yield after column chromatography.

**Figure 4:**
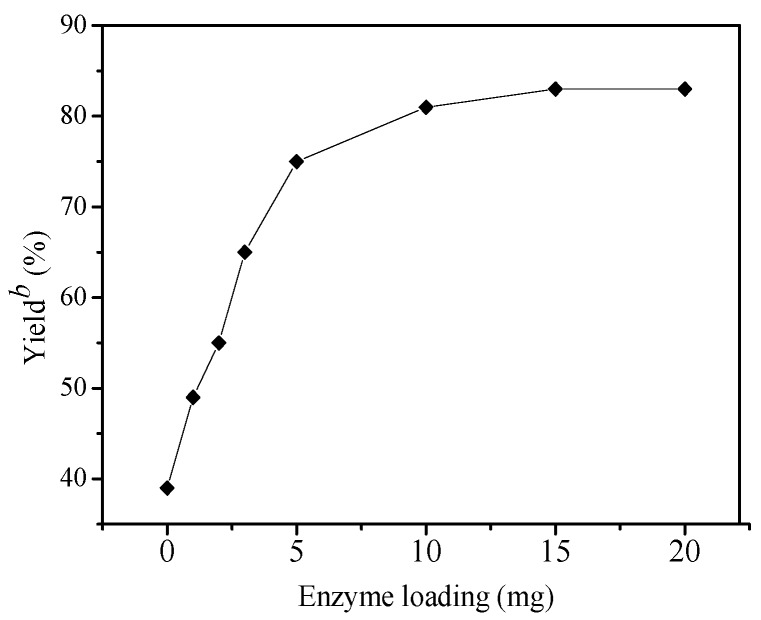
Reaction yields at different enzyme loadings ^a^. ^a^ Reaction conditions: 2-aminoacetophenone (0.3 mmol), ethyl acetoacetate (0.36 mmol), and α-chymotrypsin (0, 1, 2, 3, 5, 10, 15, 20 mg/mL) in 20% ([EMIM][BF_4_]/(H_2_O + [EMIM][BF_4_]), *v*/*v*) IL aqueous solution at 55 °C for 24 h. ^b^ Isolated yield after column chromatography.

In our previous work, heating at 60 °C contributed to superior yields; hence, the above-mentioned reaction was performed at 60 °C. Since the reaction in the 20% IL aqueous solution presented a superior trend in the yields, we decided to verify whether a lower temperature could afford excellent results in IL aqueous solution. Thus, we performed the model reaction at six different temperatures ranging from 40 to 70 °C for 24 h (Figure 3). It can be seen that the enzyme activity was lower at lower temperatures (40 °C and 45 °C). At 55 °C, the reaction gave superior results, with yields of up to 82% as compared with our previous work, where the temperature was set at 60 °C. From this, we can see that the IL aqueous solution had worked, and the result had reached our requirement. Although temperatures of 60 °C and 70 °C contributed to superior results, as a higher temperature and IL [26] can also promote this reaction (in Figure 4, when the enzyme loading was 0 mg), the key role of the enzyme as a catalyst in the reaction was not obvious in this case, and the enzyme easily reduced its activity at higher temperature. On the other hand, the energy consumption, too, had to be considered. Thus, implementing the reaction at 55 °C was the best strategy in an IL aqueous solution.

Encouraged by the above results, we further investigated the enzyme loading for enhanced results (Figure 4). The yields were improved greatly by increasing the enzyme loading. The reaction in the IL aqueous solution without any enzyme afforded a low yield, whereas a yield of 81% was obtained when 10 mg of α-chymotrypsin was loaded at 55 °C. When the enzyme loading was increased from 10 to 20 mg, only a small increase in the yield was observed. This trend may be due to the fact that the system with the IL hindered efficient dispersion of the enzyme and reduced the mass transfer rate of the substrate [27]. In addition, considering the problem of energy consumption, 10 mg was chosen as the optimum catalyst dosage for the reaction.

Under the optimized reaction conditions, the generality and scope of the α-chymotrypsin-catalyzed Friedländer condensation between 2-aminoaryl ketones and a number of α-methylene ketones were examined; the results are summarized in Table 1. A wide range of substrates could participate in the condensation reaction to give the corresponding products in moderate to excellent yields in the biphasic [BMIM][BF_4_]*/*H_2_O medium. The corresponding quinoline derivatives were synthesized in a superior way when using 2-aminobenzophenone (Table 1, Entries 8–14) compared to those obtained with 2-aminoacetophenone (Table 1, Entries 1–7). In particular, 2-aminoaryl ketones and the α-methylene ketones with a reactive α-methylene group provided the desired products in higher yields. However, in the case of the cyclic single ketone, which could not participate efficiently in the reaction, the yields were lower than 50% (Table 1, Entries 4–6, 11–12). The low yields were probably due to the substantial steric hindrance, which retarded the reaction.

## 3. Experimental

### 3.1. General Information

All chemicals were purchased from commercial suppliers and used without further purification. α-Chymotrypsin was obtained from Sigma-Aldrich (Shanghai, China). All ILs were purchased from Shanghai ChengJie Chemical Co. Ltd. (Shanghai, China).

^1^H-NMR and ^13^C-NMR spectra were recorded on Bruker AVANCE III HD 500 (Fällanden, Switzerland) and Bruker AV400 spectrometer, respectively, using CDCl_3_ as solvent. Chemical shifts (*δ*) were expressed in ppm with TMS as the internal standard, and coupling constants (*J*) were reported in Hz. Melting points were measured using a WRS-1B Digital Melting Point Apparatus (Shanghai, China).

### 3.2. General Procedure for the Synthesis of Bis(Indolyl)Methane

A mixture of 2-aminoaryl ketone (0.3 mmol, 1 equiv), α-methylene ketone (0.36 mmol, 1.2 equiv), and α-chymotrypsin (10 mg) in 1 mL of 20% IL aqueous solution (20%, [EMIM][BF_4_]/([EMIM][BF_4_] + H_2_O), *v*/*v*) was incubated in a constant temperature shaker (55 °C, 260 r/min, 24 h). The process was monitored by thin layer chromatography (TLC). After completion of the reaction, the product was extracted with 5 mL × 3 EtOAc. Then, the combined organic layer was concentrated under reduced pressure to afford the crude product, which was purified by silica gel column chromatography (PE/EtOAc = 10:1) to yield the pure product. All products were known compounds that were characterized by ^1^H-NMR.

## 4 Conclusions

In conclusion, we had successfully demonstrated an efficient approach for the synthesis of quinoline derivatives via Friedländer condensation using α-chymotrypsin as an efficient, environmentally friendly catalyst in an IL with moderate water content. The methodology developed required a low reaction temperature and reduced enzyme loading, and afforded excellent yields. A series of substrates was investigated, and the results were found to be better than those obtained with organic solvents. Importantly, the proposed methodology avoids the use of hazardous acids or bases and harsh reaction conditions, thereby stimulating the development of a green synthetic methodology. This method not only extends the application of proteases as non-specific biocatalysts, but also confirms the potential use of ILs as better alternatives to organic solvents for the Friedländer condensation between a 2-aminoaryl ketone and an α-methylene ketone.

## Figures and Tables

**Table 1:**
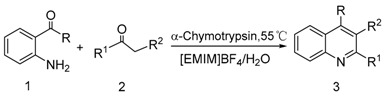
Substrate scope of α-chymotrypsin-catalyzed Friedländer condensation in an IL aqueous solution ^a^.

Entry	2-Aminoaryl Ketone (1)	α-Methylene Ketone (2)	Product (3)	Yield ^b^/%
1	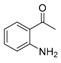	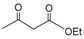	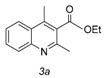	81
2	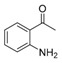	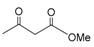	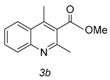	83
3	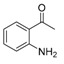	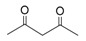	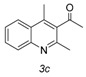	91
4	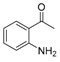	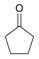	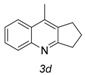	45
5	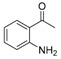	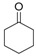	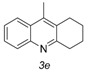	40
6	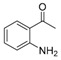	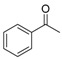	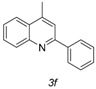	49
7	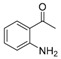	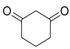	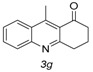	74
8	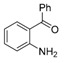	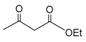	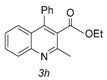	90
9	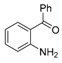	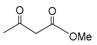	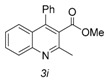	90
10	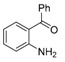	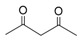	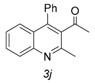	94
11	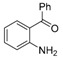	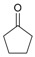	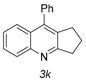	48
12	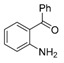	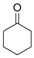	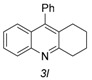	42
13	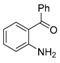	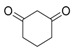	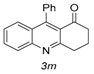	94
14	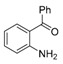	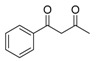	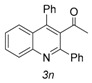	80

^a^ Reaction conditions: 2-aminoaryl ketones (0.3 mmol), α-methylene ketone (0.36 mmol), and α-chymotrypsin (10 mg) in 20% ([EMIM][BF_4_]/(H_2_O + [EMIM][BF_4_]), *v*/*v*) IL aqueous solution at 55 °C for 24 h. ^b^ Isolated yield after column chromatography.

## Supplementary Materials

Supplementary File 1
