# Supplementary material for: Ionic Liquid as an Efficient Medium for the Synthesis of Quinoline Derivatives via α-Chymotrypsin-Catalyzed Friedländer Condensation
Source: Molecules. 2017 May 8;22(5):762. doi: 10.3390/molecules22050762 (PMC6154722; doi:10.3390/molecules22050762)

# Supplementary information

## Ionic liquid as an efficient medium for the synthesis of quinoline derivatives via α-chymotrypsin-catalyzed Friedlander condensation

Zhang-Gao Le[1,2], Meng Liang[2], Zhong-Sheng Chen[2], Sui-Hong Zhang[2], Zong-Bo Xie*[1,2]

*1 Jiangxi 2011 Joint Center for the Innovative Mass Spectrometry and Instrumentation, East China University of Technology, Nanchang 330013, People's Republic of China*

*2 School of Chemistry, Biology and Material Science, East China University of Technology, Nanchang 330013, People's Republic of China*

E-mail: zhgle@ecit.edu.cn (Z.-G Le), zbxie@ecit.edu.cn (Z.-B. Xie).

**Table of Contents**

Spectroscopic data of the products .................................................................... 2

$^{1}$H NMR and $^{13}$C NMR Spectroscopic data ....................................................... 5

## Spectroscopic data of products

**Ethyl-2,4-dimethylquinoline-3-carboxylate** (**3a**)

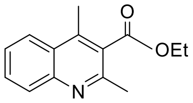

Yellow oil, yield 81%; $^1$H NMR (500 MHz, $CDCl_3$) δ 8.02 (d, *J* = 8.4 Hz, 1H), 7.98 (d, *J* = 8.3 Hz, 1H), 7.70 (t, *J* = 7.6 Hz, 1H), 7.53 (t, *J* = 7.6 Hz, 1H), 4.49 (q, *J* = 7.0 Hz, 2H), 2.71 (s, 3H), 2.65 (s, 3H), 1.44 (t, *J* = 7.1 Hz, 3H); $^{13}$C NMR (101 MHz, $CDCl_3$) δ 169.18, 154.32, 147.07, 141.45, 130.05, 129.24, 127.99, 126.31, 125.77, 123.98, 61.66, 23.77, 15.68, 14.24.

**Methyl 2,4-dimethyl quinoline-3-carboxylate (3b)**

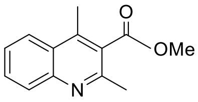

Yellow oil, yield 83%; $^1$H NMR (500 MHz, $CDCl_3$) δ 8.02 (d, *J* = 8.3 Hz, 1H), 7.97 (d, *J* = 7.9 Hz, 1H), 7.71 (t, *J* = 7.0 Hz, 1H), 7.53 (s, 1H), 4.00 (s, 3H), 2.70 (s, 3H), 2.63 (s, 3H); $^{13}$C NMR (101 MHz, $CDCl_3$) δ 169.68, 154.36, 147.12, 141.69, 130.14, 129.26, 127.71, 126.35, 125.71, 124.02, 52.51, 23.83, 15.82.

**1-(2,4-Dimethylquinolin-3-yl)ethanone (3c)**

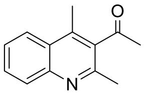

Yellow oil, yield 91%; $^1$H NMR (500 MHz, $CDCl_3$) δ 8.02 (d, *J* = 8.4 Hz, 1H), 7.97 (d, *J* = 8.3 Hz, 1H), 7.71 (t, *J* = 7.5 Hz, 1H), 7.54 (t, *J* = 7.5 Hz, 1H), 2.64 (s, 3H), 2.58 (d, *J* = 7.2 Hz, 6H). $^{13}$C NMR (101 MHz, $CDCl_3$) δ 206.66, 152.61, 146.89, 138.68, 135.73, 129.87, 129.23, 126.43, 125.98, 123.68, 32.68, 23.54, 15.27.

**9-Methyl-2,3-dihydro-1*H*-cyclopenta[*b*]quinolone (3d)**

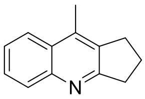

White solid, yield 45%, m.p. 56-60ºC; $^1$H NMR (500 MHz, $CDCl_3$) δ 8.01 (d, *J* = 8.3 Hz, 1H), 7.92 (d, *J* = 8.3 Hz, 1H), 7.61 (t, *J* = 7.5 Hz, 1H), 7.47 (t, *J* = 7.5 Hz, 1H), 3.16 (t, *J* = 7.5 Hz, 2H), 3.04 (t, *J* = 7.2 Hz, 2H), 2.57 (s, 3H), 2.23 – 2.13 (m, 2H); $^{13}$C NMR (101 MHz, $CDCl_3$) δ 166.94, 147.38, 138.13, 134.02, 129.11, 128.00, 127.05, 125.25, 123.34, 35.10, 29.64, 22.98, 14.89.

**9-Methyl-1,2,3,4-tetrahydroacridine (3e)**

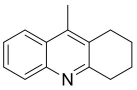

White solid, yield 40%, m.p. 73-77 ºC; $^1$H NMR (500 MHz, $CDCl_3$) δ 8.01 (d, *J* = 8.4 Hz, 1H), 7.98 (d, *J* = 8.5 Hz, 1H), 7.62 (t, *J* = 7.5 Hz, 1H), 7.47 (t, *J* = 7.5 Hz, 1H), 3.15 (s, 2H), 2.91 (s, 2H), 2.57 (s, 3H), 1.94 (s, 4H); $^{13}$C NMR (101 MHz, $CDCl_3$) δ 158.50, 145.67, 141.58, 130.93, 128.78, 128.24,

126.94, 125.34, 123.32, 34.36, 27.09, 23.19, 22.73, 13.58.

**4-Methyl-2-phenylquinoline (3f)**

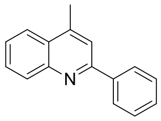

Yellow oil, yield 49%; $^1$H NMR (500 MHz, $CDCl_3$) δ 8.24 (d, *J* = 7.6 Hz, 1H), 8.15 (d, *J* = 7.4 Hz, 2H), 8.01 (d, *J* = 8.3 Hz, 1H), 7.74 (d, *J* = 11.7 Hz, 2H), 7.59 – 7.44 (m, 4H), 2.79 (s, 3H); $^{13}$C NMR (101 MHz, $CDCl_3$) δ 157.02, 147.79, 145.27, 139.49, 130.03, 129.54, 129.36, 128.83, 127.66, 127.27, 126.18, 123.65, 119.89, 19.10.

**9-Methyl -3,4-dihydroacridin-1(2*H*)-one (3g)**

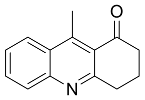

White solid, yield 74%, m.p. 62-65 °C; $^1$H NMR (500 MHz, $CDCl_3$) δ 8.21 (d, *J* = 8.5 Hz, 1H), 8.02 (d, *J* = 8.3 Hz, 1H), 7.78 (t, *J* = 7.5 Hz, 1H), 7.57 (t, *J* = 7.6 Hz, 1H), 3.28 (t, *J* = 5.9 Hz, 2H), 3.05 (s, 3H), 2.81 (t, *J* = 6.4 Hz, 2H), 2.26 – 2.18 (m, 2H); $^{13}$C NMR (101 MHz, $CDCl_3$) δ 199.96, 161.54, 149.46, 147.24, 130.99, 128.54, 127.13, 125.83, 124.92, 124.81, 40.54, 34.17, 20.79, 15.51.

**Ethyl-2-methyl-4-phenylquinoline-3-carboxylate (3h)**

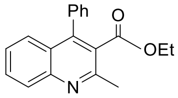

White solid, yield 90%, m.p. 98-100 °C; $^1$H NMR (500 MHz, $CDCl_3$) δ 8.10 (d, *J* = 8.4 Hz, 1H), 7.73 (t, *J* = 7.5 Hz, 1H), 7.59 (d, *J* = 8.3 Hz, 1H), 7.48 (s, 3H), 7.44 (t, *J* = 7.6 Hz, 1H), 7.37 (s, 2H), 4.07 (q, *J* = 7.0 Hz, 2H), 2.80 (s, 3H), 0.95 (t, *J* = 7.0 Hz, 3H); $^{13}$C NMR (101 MHz, $CDCl_3$) δ 168.45, 154.73, 147.67, 146.32, 135.75, 130.29, 129.39, 128.84, 128.47, 128.24, 127.44, 126.52, 126.44, 125.17, 61.35, 23.81, 13.65.

**Methyl-2-methyl-4-phenylquinoline-3-carboxylate (3i)**

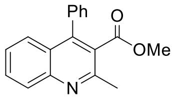

White solid, yield 90%, m.p. 99-100 °C; $^1$H NMR (500 MHz, $CDCl_3$) δ 8.09 (d, *J* = 8.4 Hz, 1H), 7.73 (t, *J* = 7.6 Hz, 1H), 7.60 (d, *J* = 8.3 Hz, 1H), 7.49 (d, *J* = 5.3 Hz, 3H), 7.44 (t, *J* = 7.7 Hz, 1H), 7.36 (d, *J* = 6.4 Hz, 2H), 3.58 (s, 3H), 2.79 (s, 3H); $^{13}$C NMR (101 MHz, $CDCl_3$) δ 169.01, 154.55, 147.75, 146.43, 135.68, 130.35, 129.25, 128.87, 128.52, 128.29, 127.29, 126.55, 126.48, 125.09, 52.18, 23.83.

**1-(2-Methyl-4-phenylquinolin-3-yl)ethanone (3j)**:

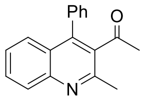

White solid, yield 94%, m.p. 114-116 °C; $^1$H NMR (500 MHz, $CDCl_3$) δ 8.09 (d, *J* = 8.3 Hz, 1H), 7.73 (t, *J* = 7.5 Hz, 1H), 7.63 (d, *J* = 8.3 Hz, 1H), 7.52 (s, 3H), 7.46 (t, *J* = 7.5 Hz, 1H), 7.37 (s, 2H), 2.71 (s, 3H), 2.01 (s, 3H); $^{13}$C NMR (101 MHz, $CDCl_3$) δ 205.72, 153.56, 147.52, 143.97, 135.21, 134.84, 130.14, 130.05, 128.93, 128.87, 128.73, 126.55, 126.18, 125.05, 31.96, 23.90.

**9-Phenyl-2,3-dihydro-1*H*-cyclopenta[*b*]quinolone (3k)**

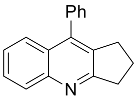


White solid, yield 48%, 138-141 ºC; $^1$H NMR (500 MHz, $CDCl_3$) δ 8.09 (d, *J* = 8.0 Hz, 1H), 7.64 (d, *J* = 7.5 Hz, 2H), 7.53 (t, *J* = 7.0 Hz, 2H), 7.48 (d, *J* = 7.1 Hz, 1H), 7.38 (t, *J* = 10.0 Hz, 3H), 3.26 (t, *J* = 7.4 Hz, 2H), 2.91 (t, *J* = 7.1 Hz, 2H), 2.21 – 2.14 (m, 2H); $^{13}$C NMR (101 MHz, $CDCl_3$) δ 167.42, 147.93, 142.74, 136.76, 133.67, 129.30, 128.81, 128.52, 128.25, 128.00, 126.23, 125.66, 125.51, 35.22, 30.35, 23.54.

**9-Phenyl-1, 2, 3, 4-tetrahydroacridine (3l)**

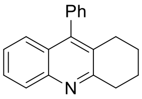


White solid, yield 42%, 139-142 ºC; $^1$H NMR (500 MHz, $CDCl_3$) δ 8.03 (d, *J* = 8.4 Hz, 1H), 7.60 (d, *J* = 2.9 Hz, 1H), 7.52 (t, *J* = 7.1 Hz, 2H), 7.49 – 7.44 (m, 1H), 7.32 (s, 2H), 7.23 (d, *J* = 7.0 Hz, 2H), 3.21 (t, *J* = 6.3 Hz, 2H), 2.61 (t, *J* = 6.2 Hz, 2H), 2.00 – 1.92 (m, 2H), 1.80 (d, *J* = 4.8 Hz, 2H); $^{13}$C NMR (101 MHz, $CDCl_3$) δ 159.09, 146.55, 146.29, 137.16, 129.13, 128.62, 128.40, 128.38, 128.37, 127.75, 126.69, 125.81, 125.40, 34.27, 28.07, 23.04, 22.93.

**9-Phenyl-3,4-dihydroacridin-1(2*H*)-one (3m)**

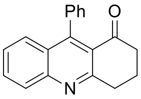


White solid, yield 94%, 154-156 ºC; $^1$H NMR (500 MHz, $CDCl_3$) δ 8.08 (d, *J* = 8.4 Hz, 1H), 7.77 (t, *J* = 7.5 Hz, 1H), 7.54 – 7.44 (m, 4H), 7.41 (t, *J* = 7.5 Hz, 1H), 7.18 (d, *J* = 6.6 Hz, 2H), 3.39 (t, *J* = 5.9 Hz, 2H), 2.71 (t, *J* = 6.3 Hz, 2H), 2.30 – 2.22 (m, 2H); $^{13}$C NMR (101 MHz, $CDCl_3$) δ 197.35, 161.76, 150.88, 148.15, 137.17, 131.24, 128.02, 127.72, 127.61, 127.06, 126.99, 125.95, 123.37, 40.15, 34.13, 20.90.

**1-(2,4-Diphenyl quinolin-3-yl)ethanone (3n)**

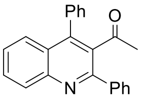


White solid, yield 80%, 136-138ºC; $^1$H NMR (500 MHz, $CDCl_3$) δ 8.15 (d, *J* = 8.4 Hz, 1H), 7.76 (t, *J* = 7.5 Hz, 1H), 7.60 (d, *J* = 7.6 Hz, 3H), 7.46 (t, *J* = 7.5 Hz, 2H), 7.33 – 7.15 (m, 7H), 2.64 (s, 3H); $^{13}$C NMR (101 MHz, $CDCl_3$) δ 197.24, 154.17, 147.26, 145.20, 136.70, 134.33, 133.07, 132.01, 129.68, 129.54, 128.77, 128.36, 127.99, 127.83, 127.56, 126.08, 125.78, 124.82, 23.52.

# $^{1}$H and $^{13}$C NMR spectra of the products

**Ethyl-2,4-dimethylquinoline-3-carboxylate (3a)**

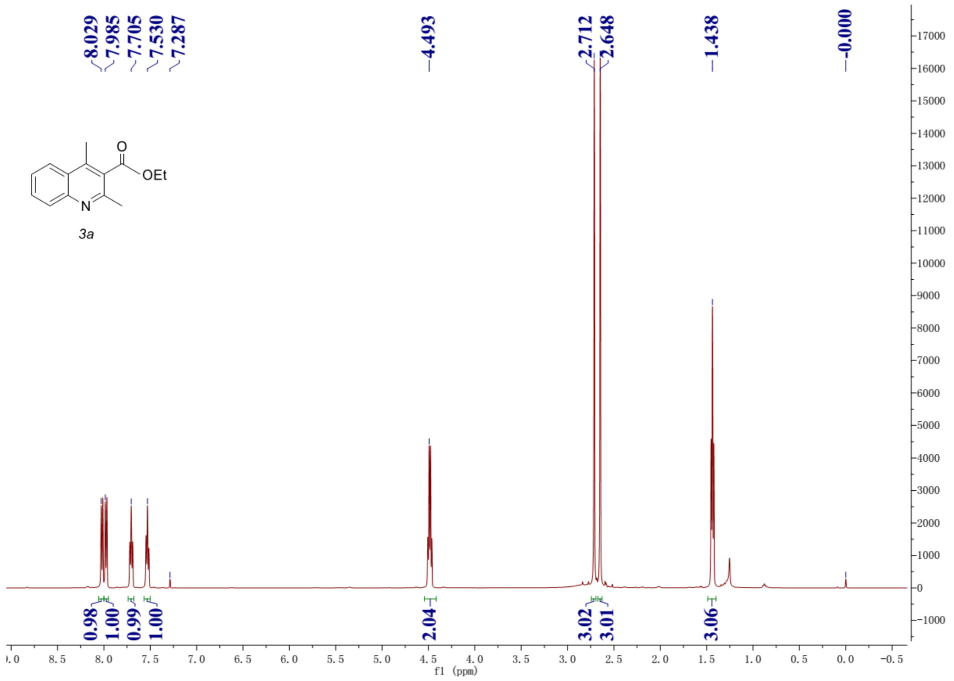


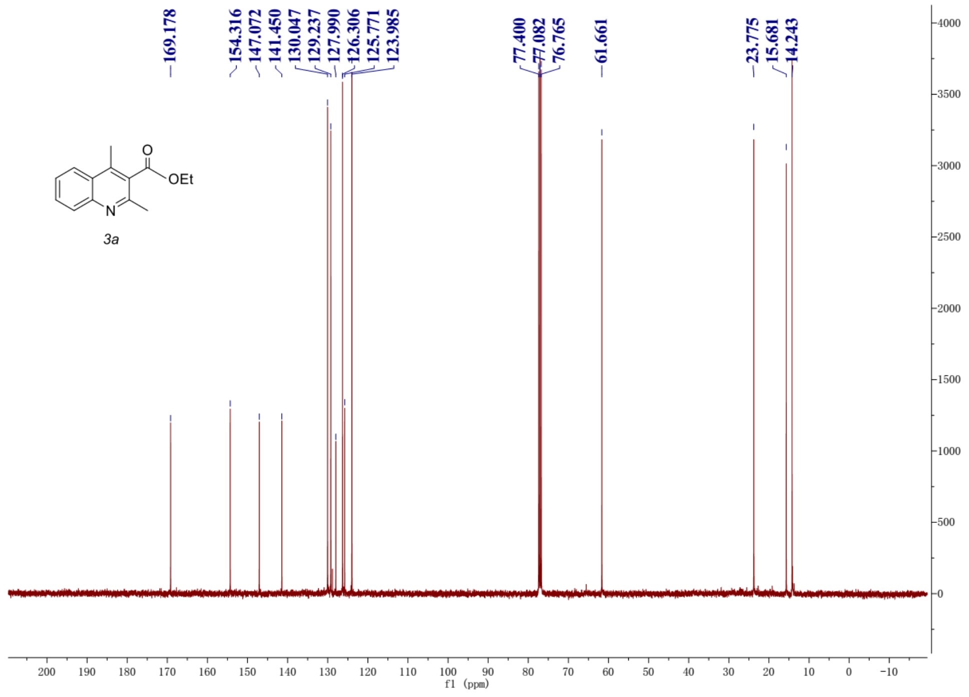

**Methyl 2,4-dimethyl quinoline-3-carboxylate (3b)**

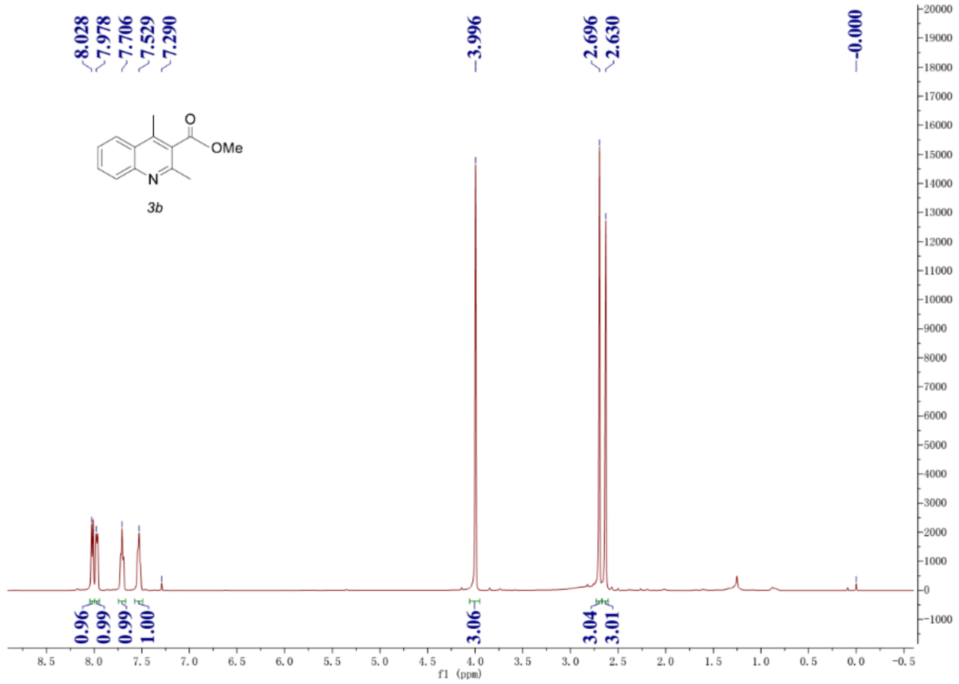


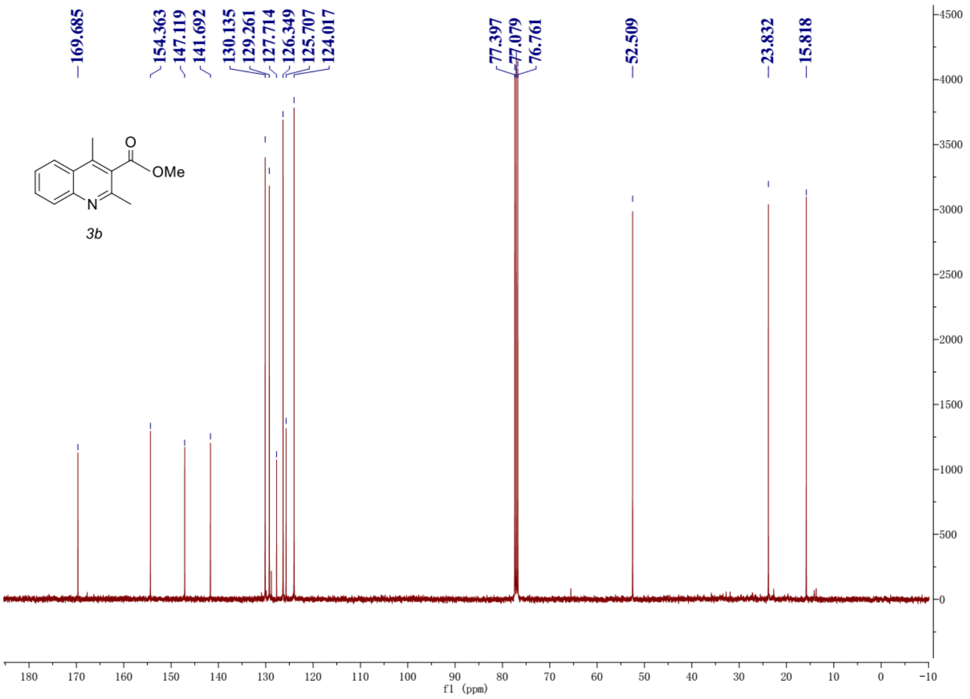

1-(2,4-Dimethylquinolin-3-yl)ethanone (3c)

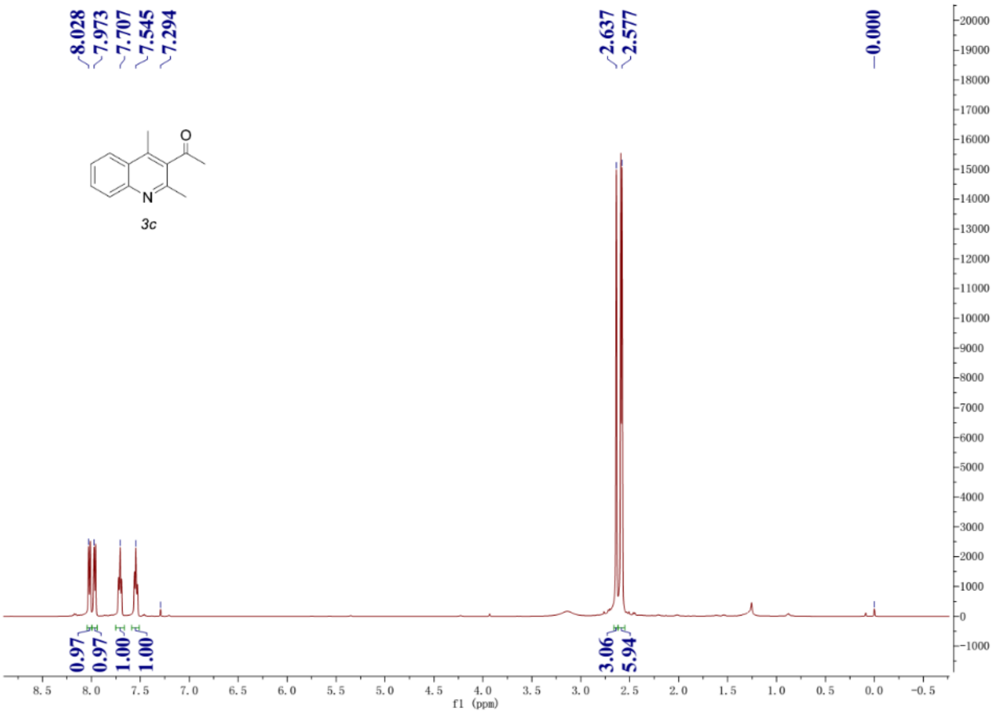


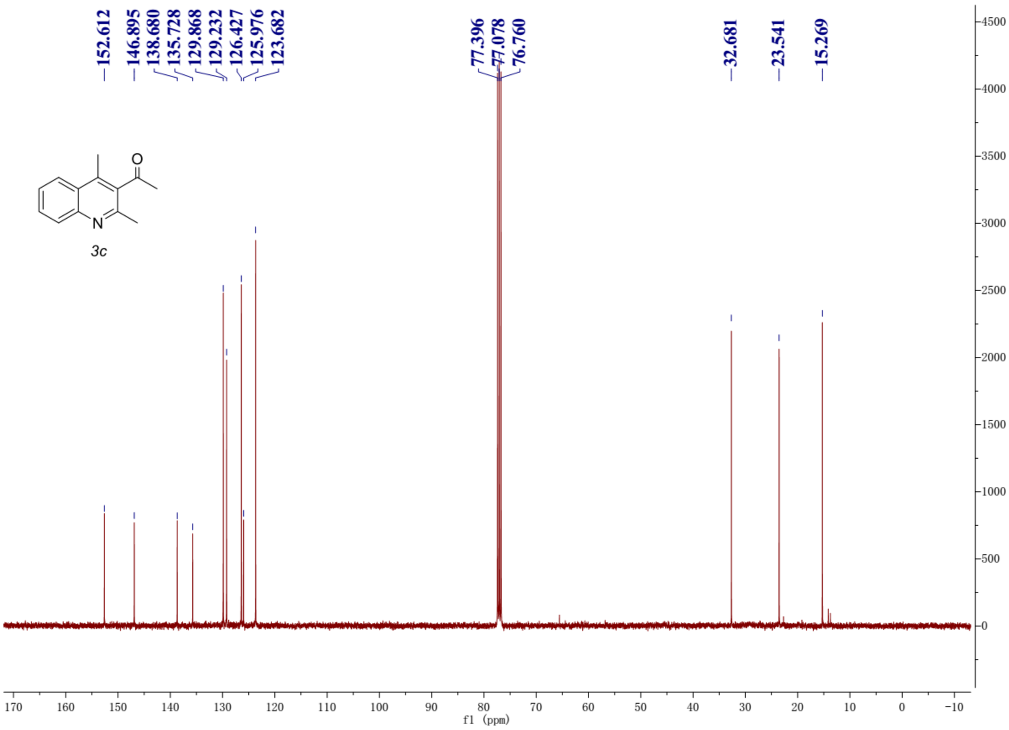

**9-Methyl-2,3-dihydro-1*H*-cyclopenta[*b*]quinolone (3d)**

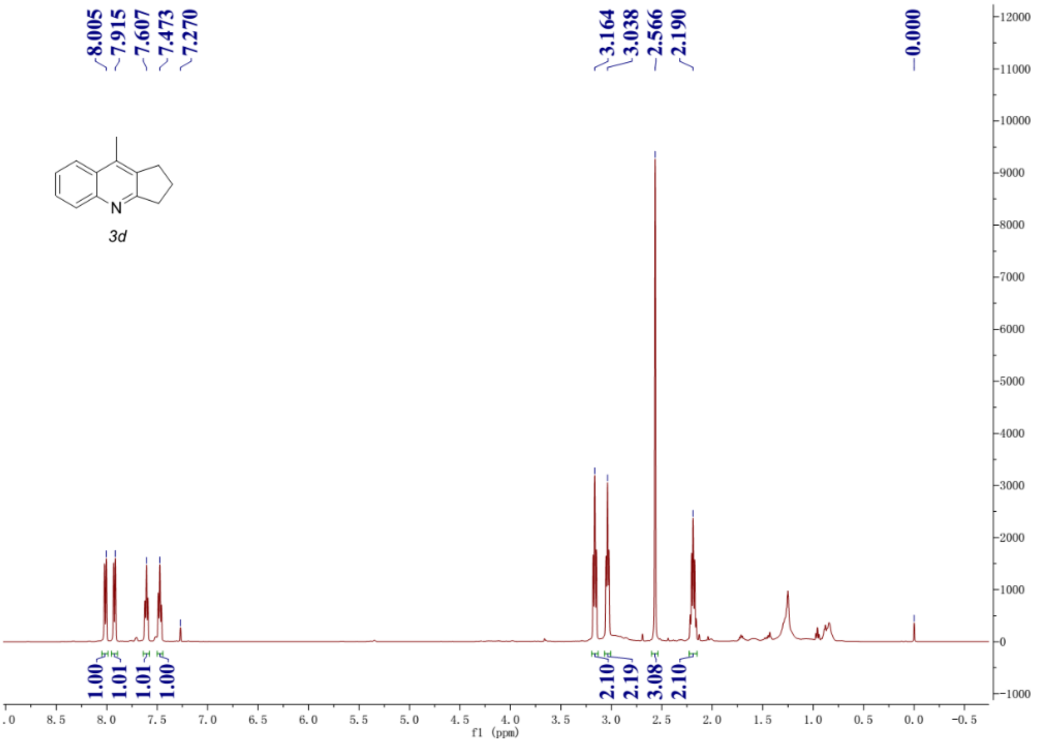


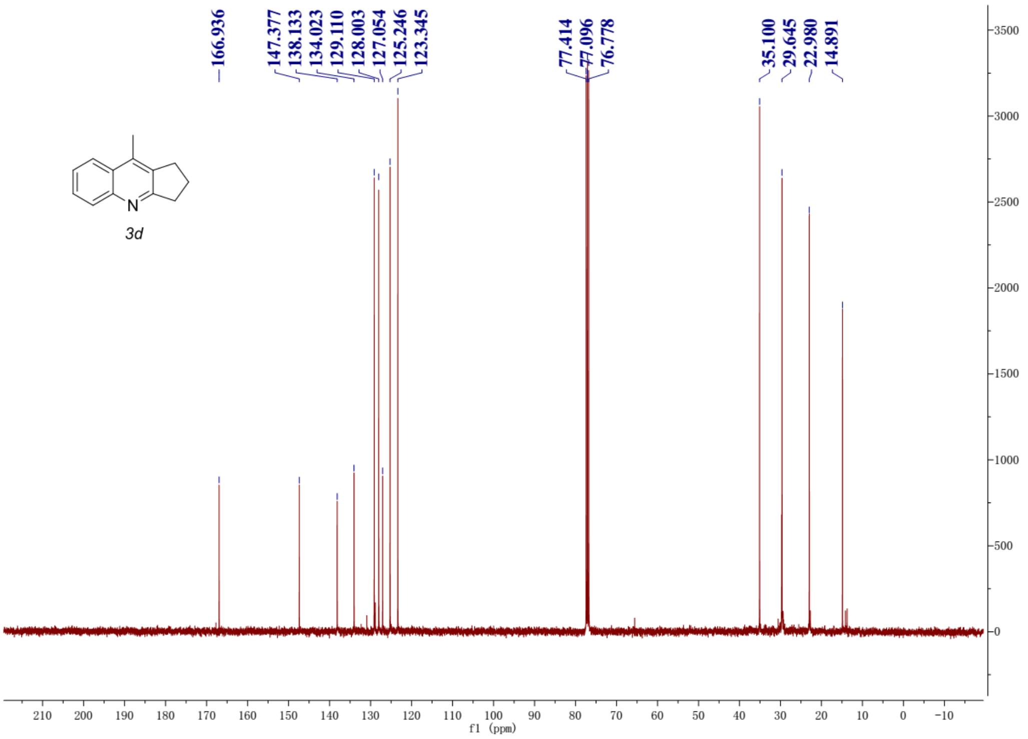

**9-Methyl-1,2,3,4-tetrahydroacridine (3e)**

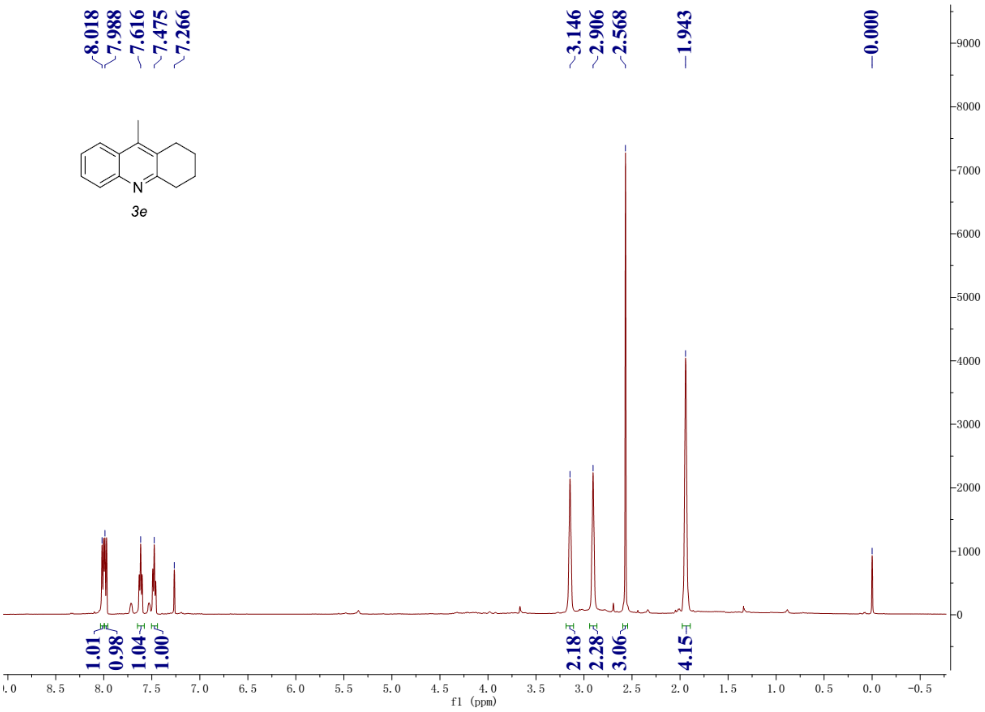


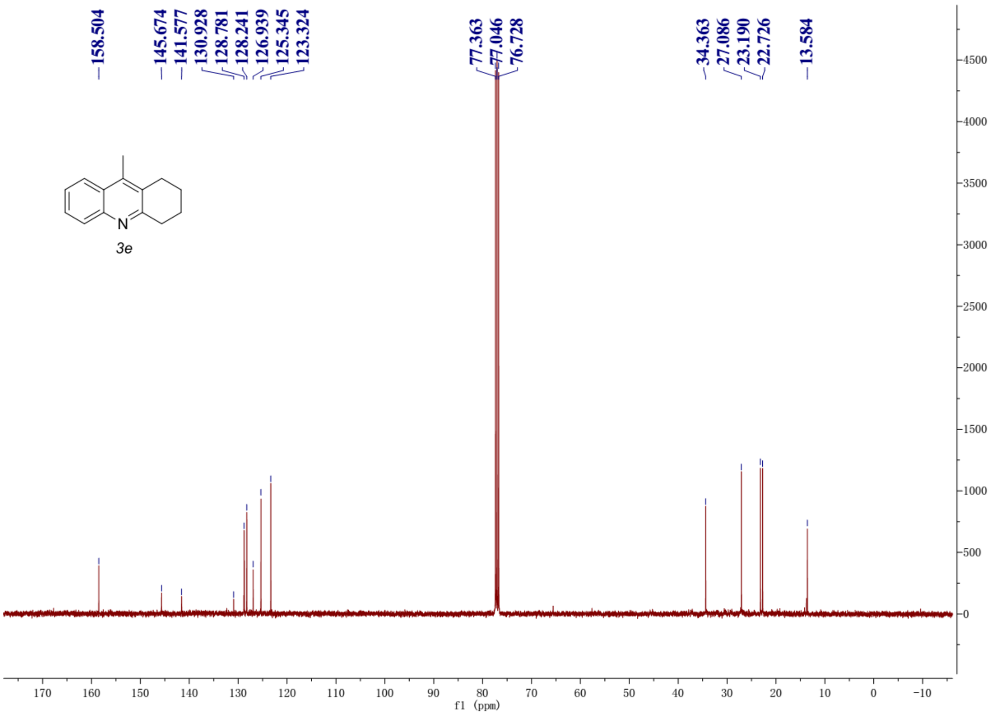

# 4-Methyl-2-phenylquinoline (3f)

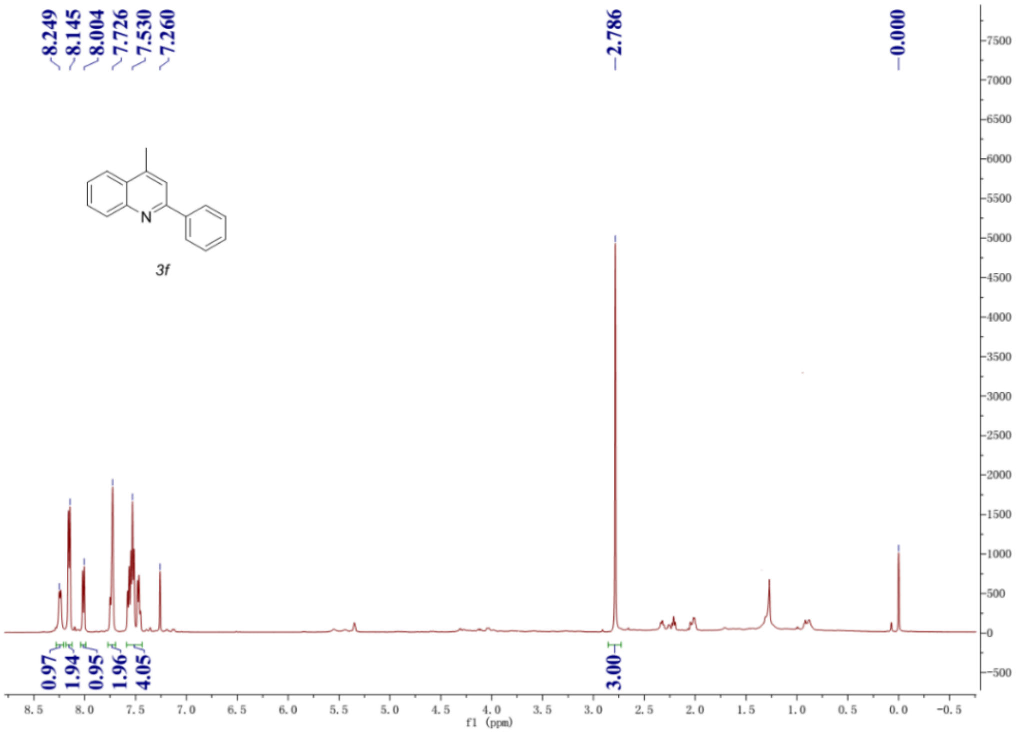


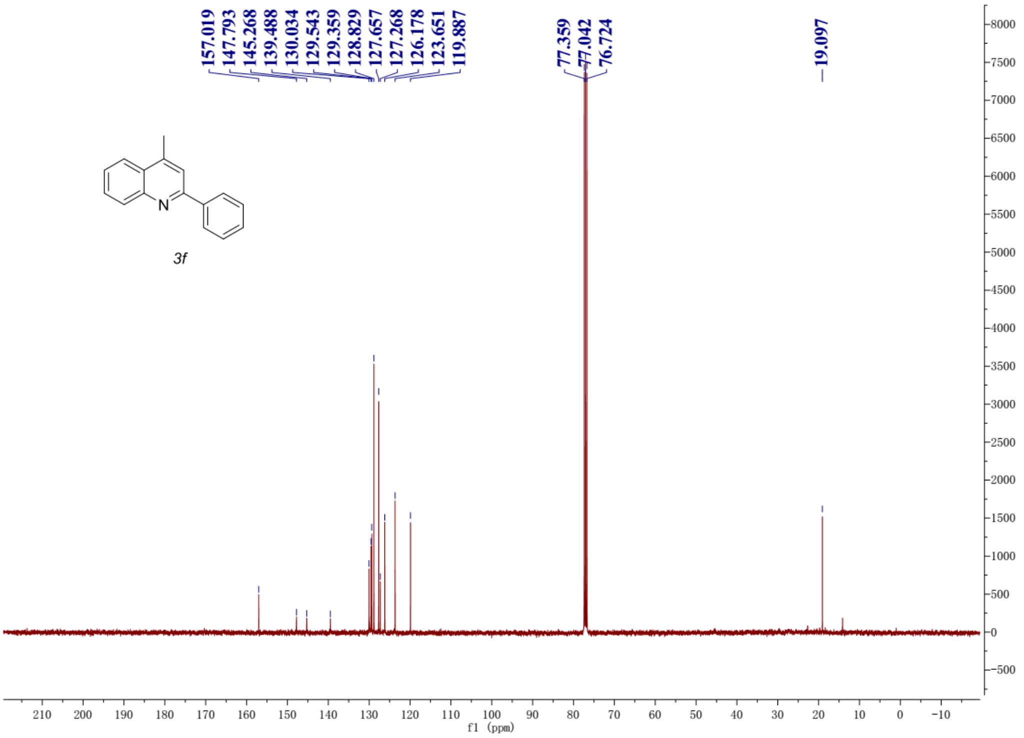

**9-Methyl -3,4-dihydroacridin-1(2*H*)-one (3g)**

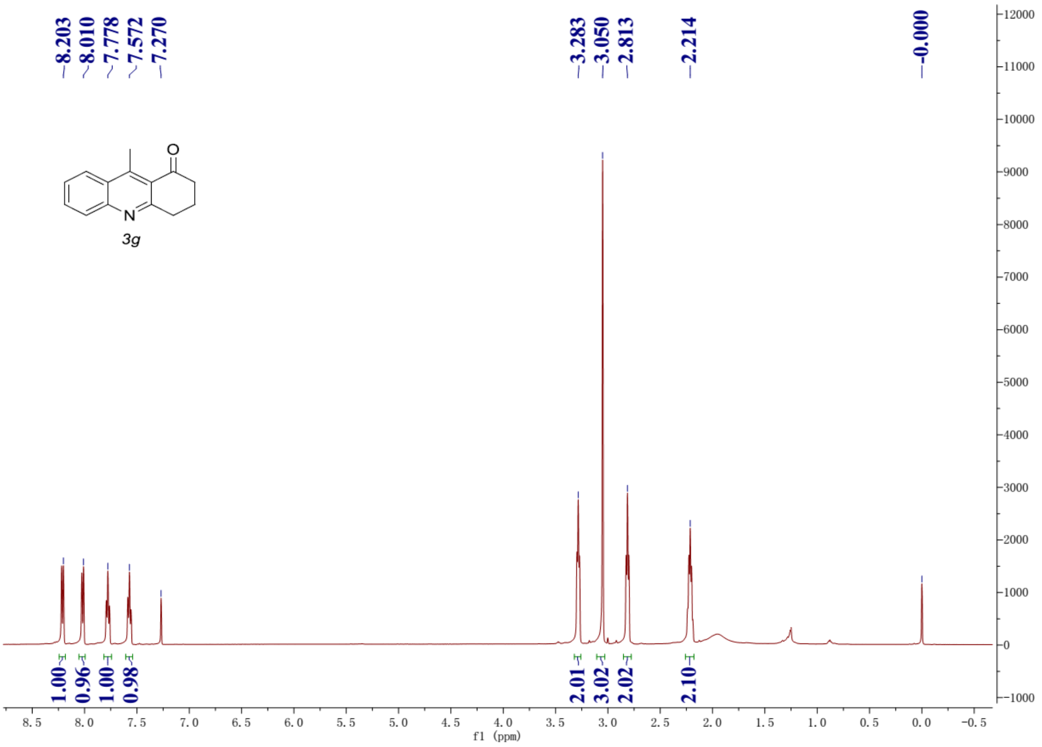


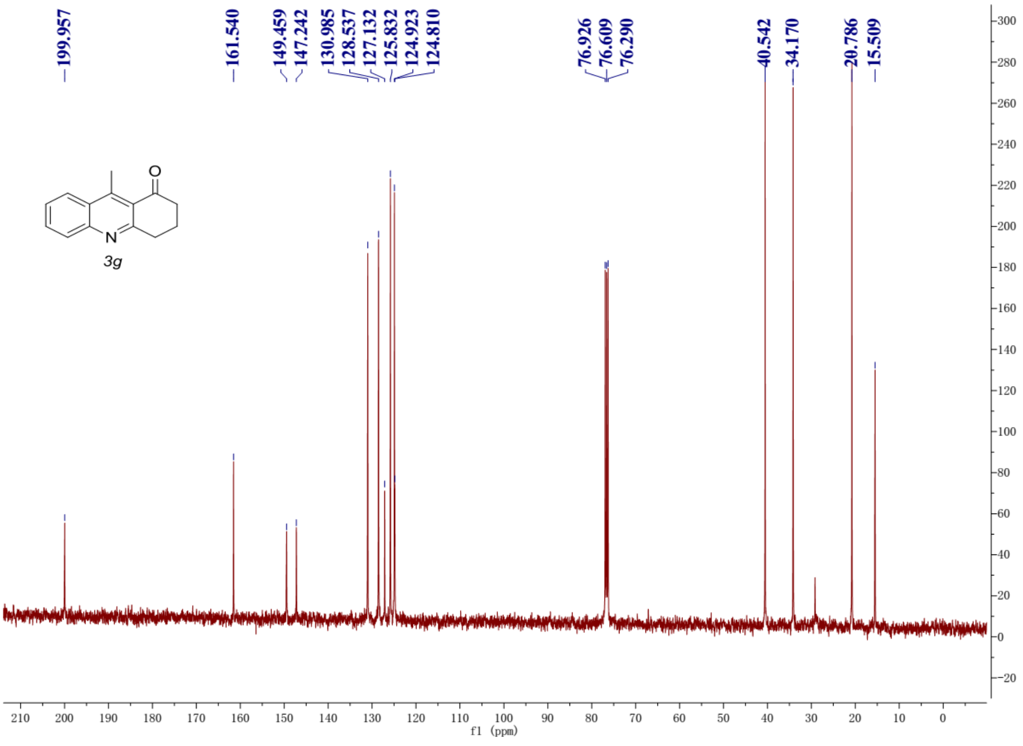

**Ethyl-2-methyl-4-phenylquinoline-3-carboxylate (3h)**

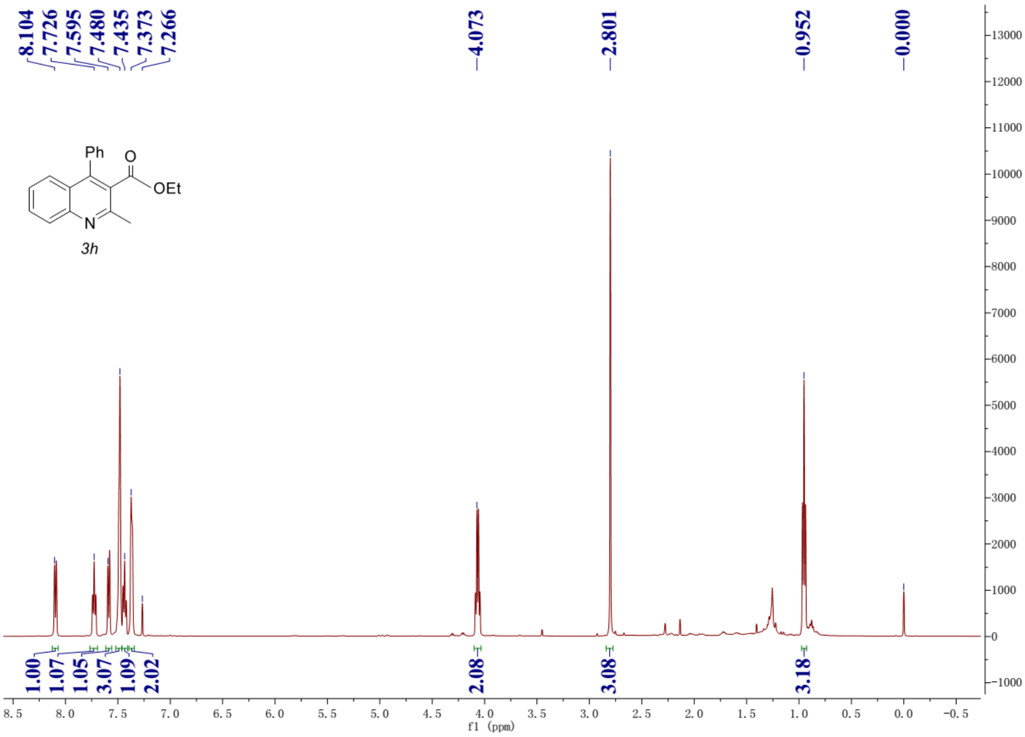


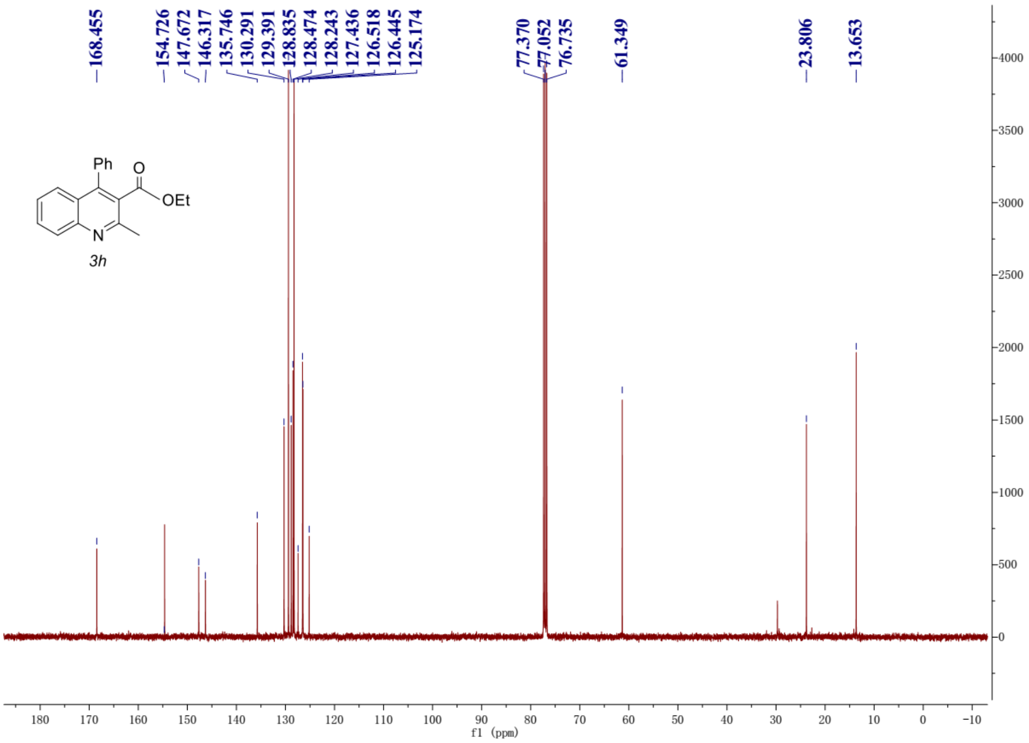

**Methyl-2-methyl-4-phenylquinoline-3-carboxylate (3i)**

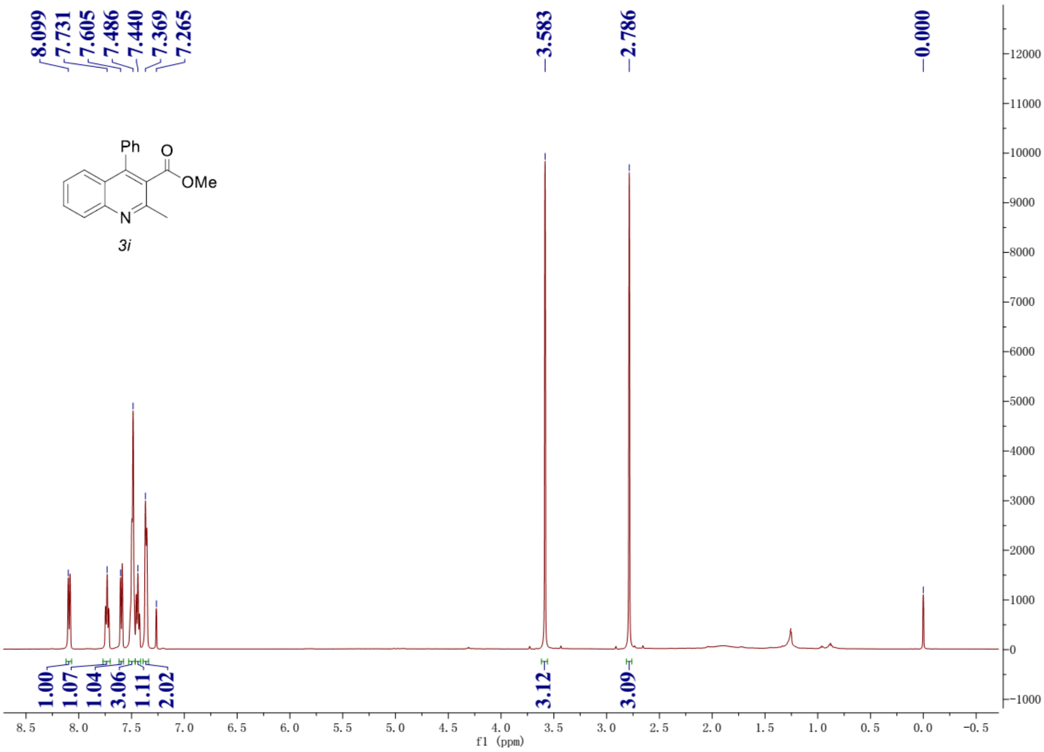

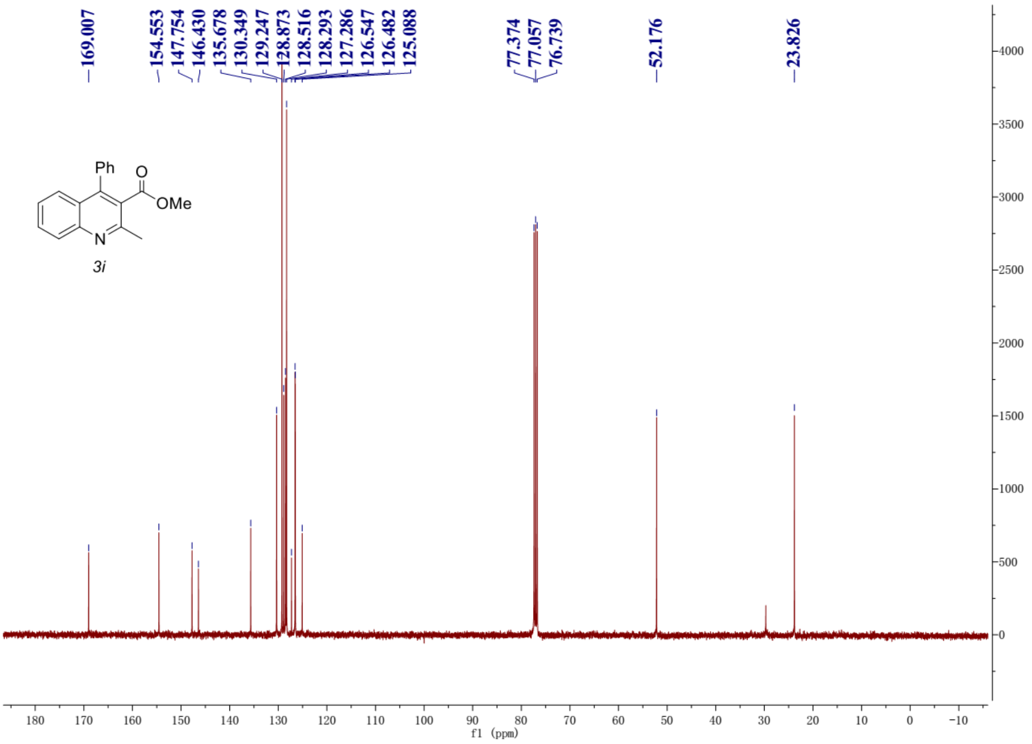

## 1-(2-Methyl-4-phenylquinolin-3-yl)ethanone (3j)

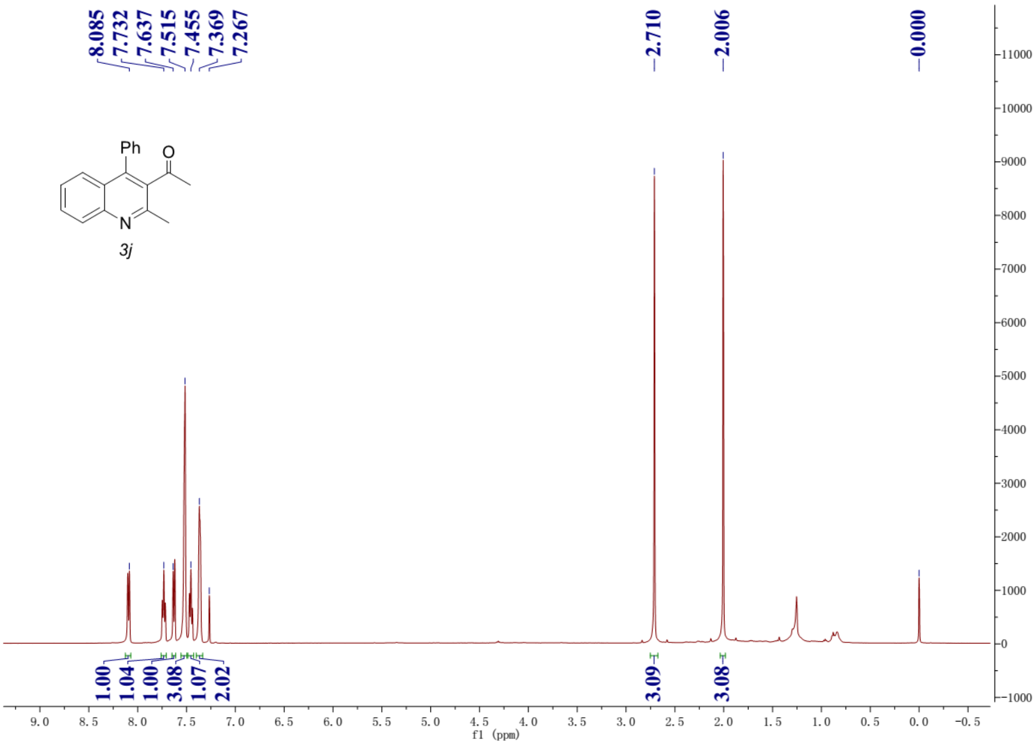


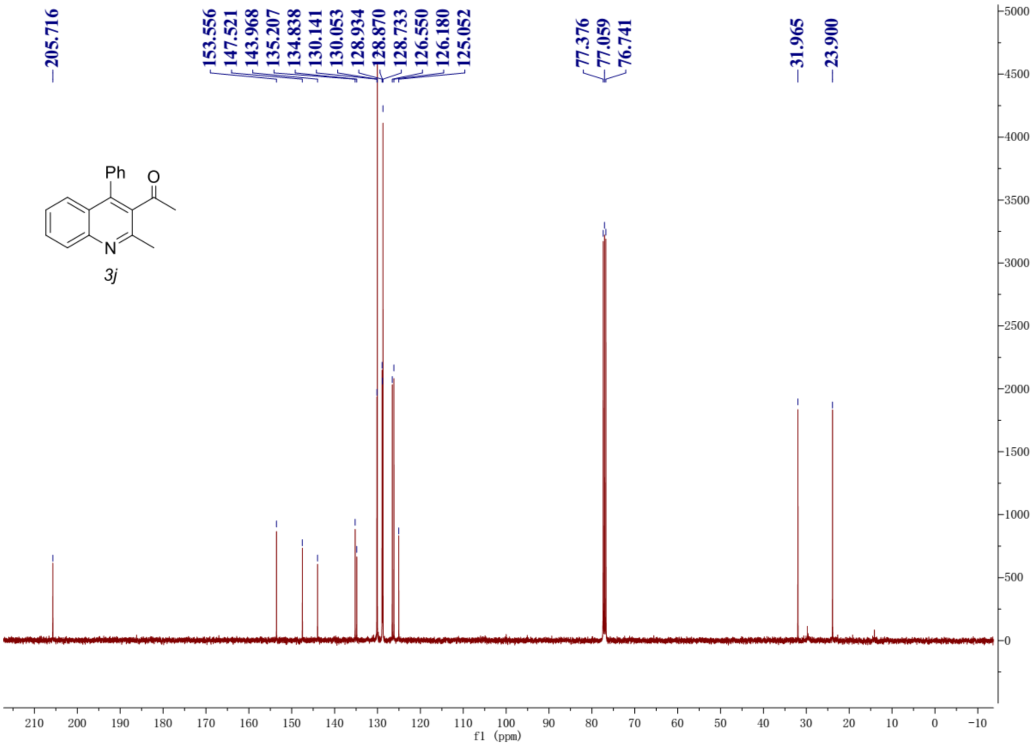

**9-Phenyl-2,3-dihydro-1*H*-cyclopenta[*b*]quinolone (3k)**

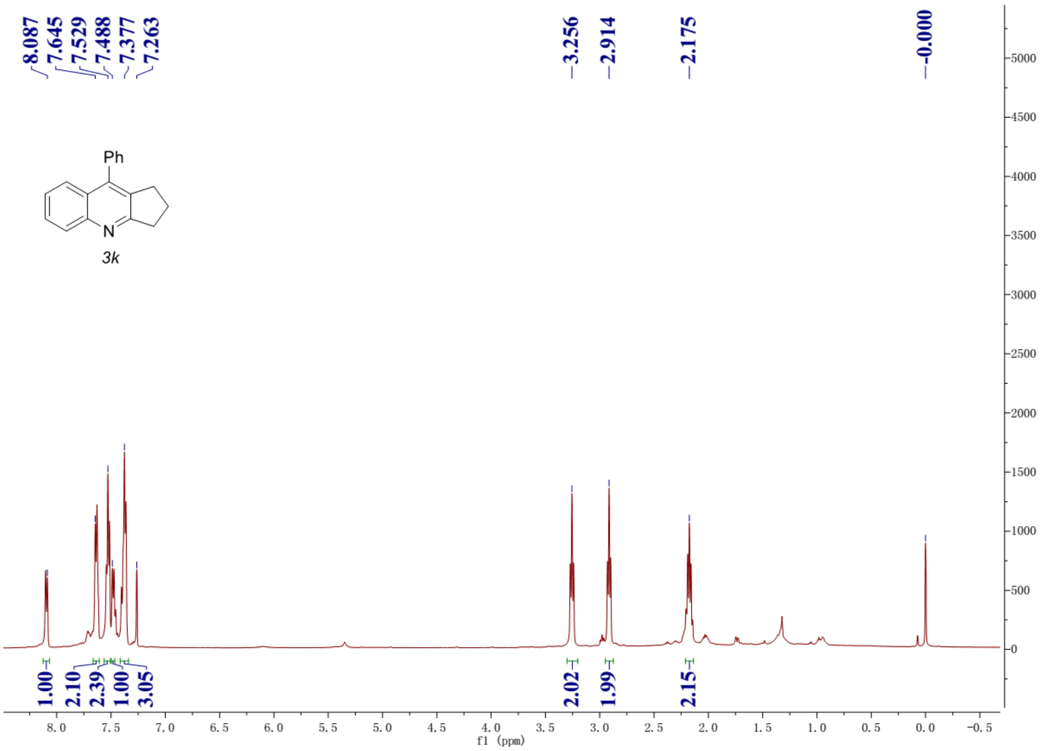


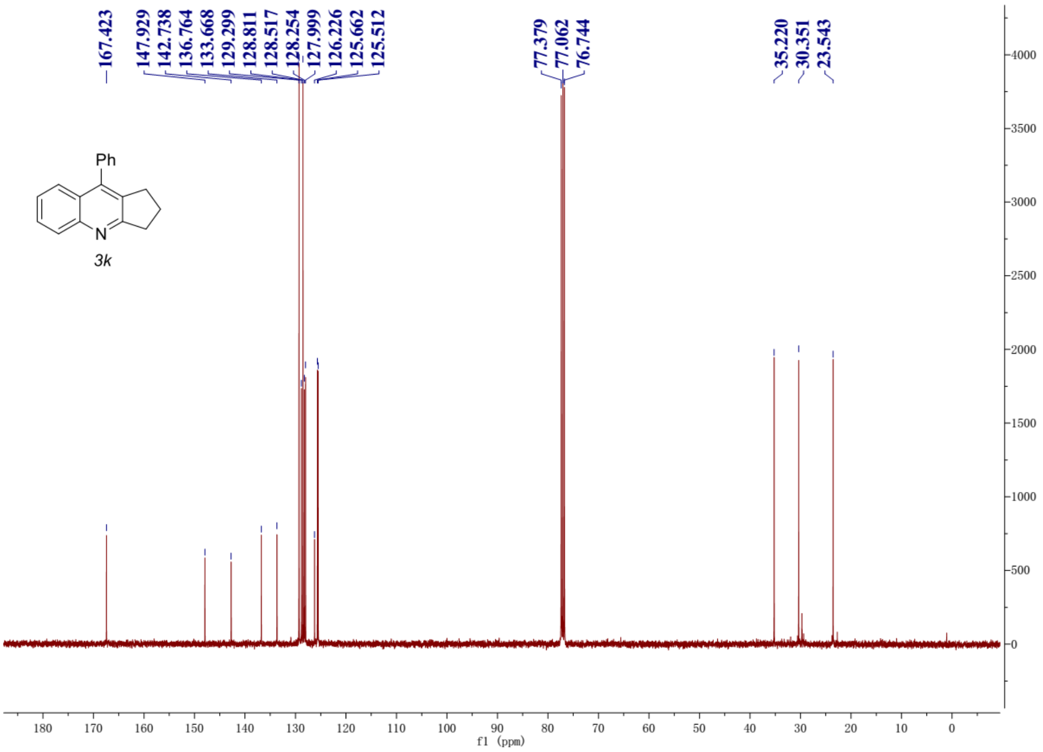

**9-Phenyl-1, 2, 3, 4-tetrahydroacridine (3l)**

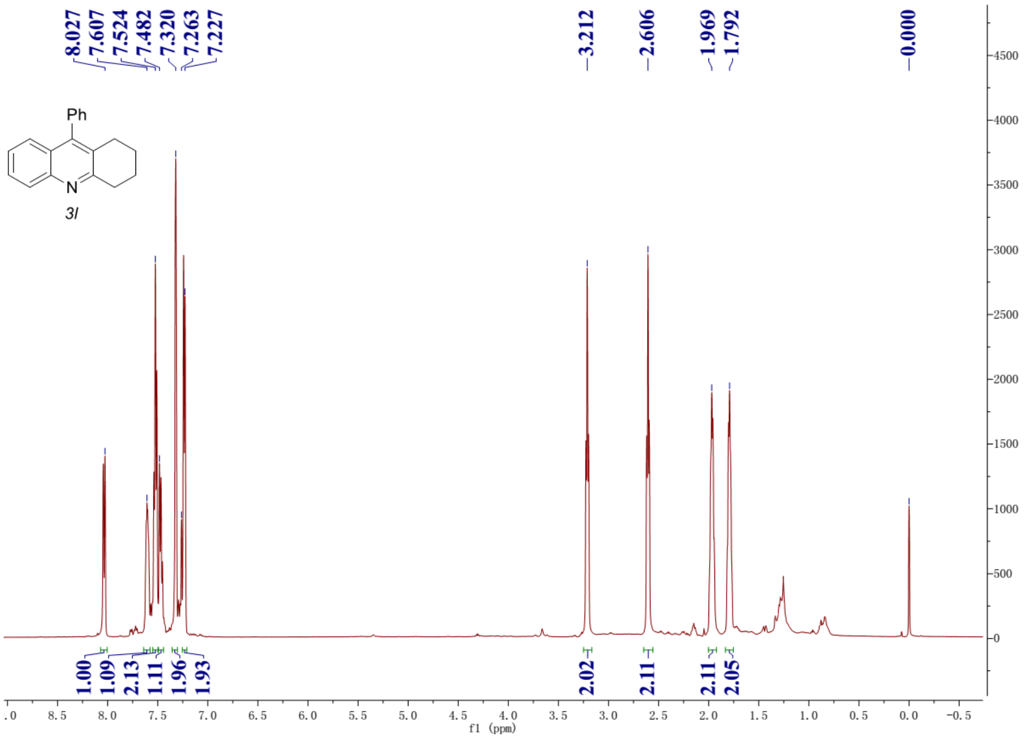


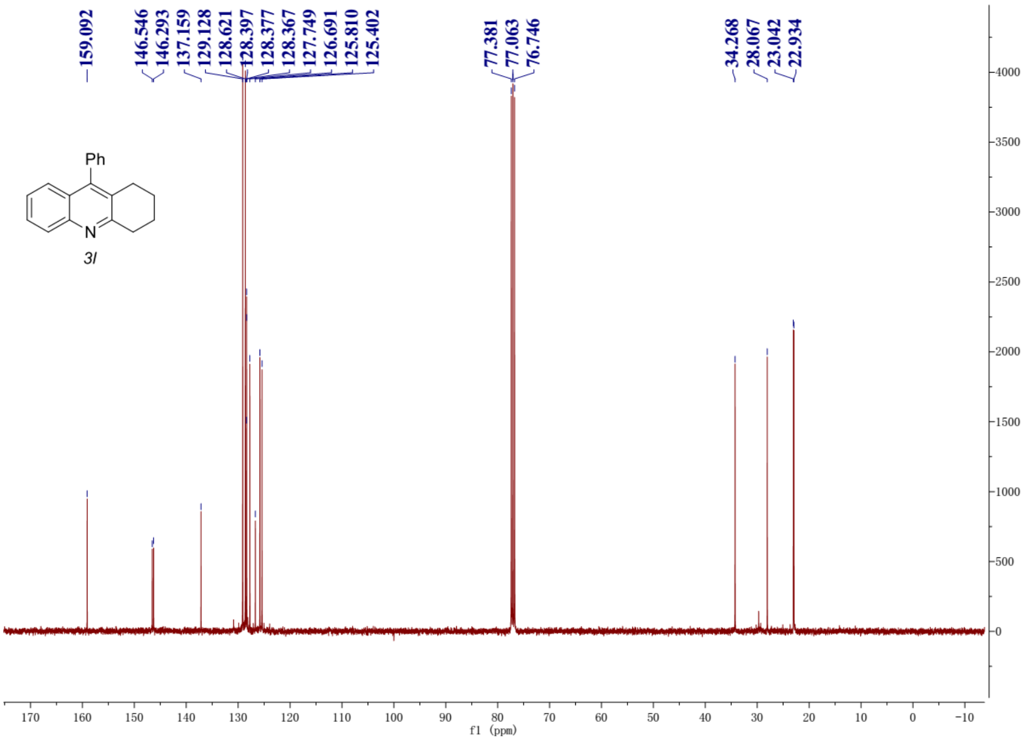

**9-Phenyl-3,4-dihydroacridin-1(2*H*)-one (3m)**

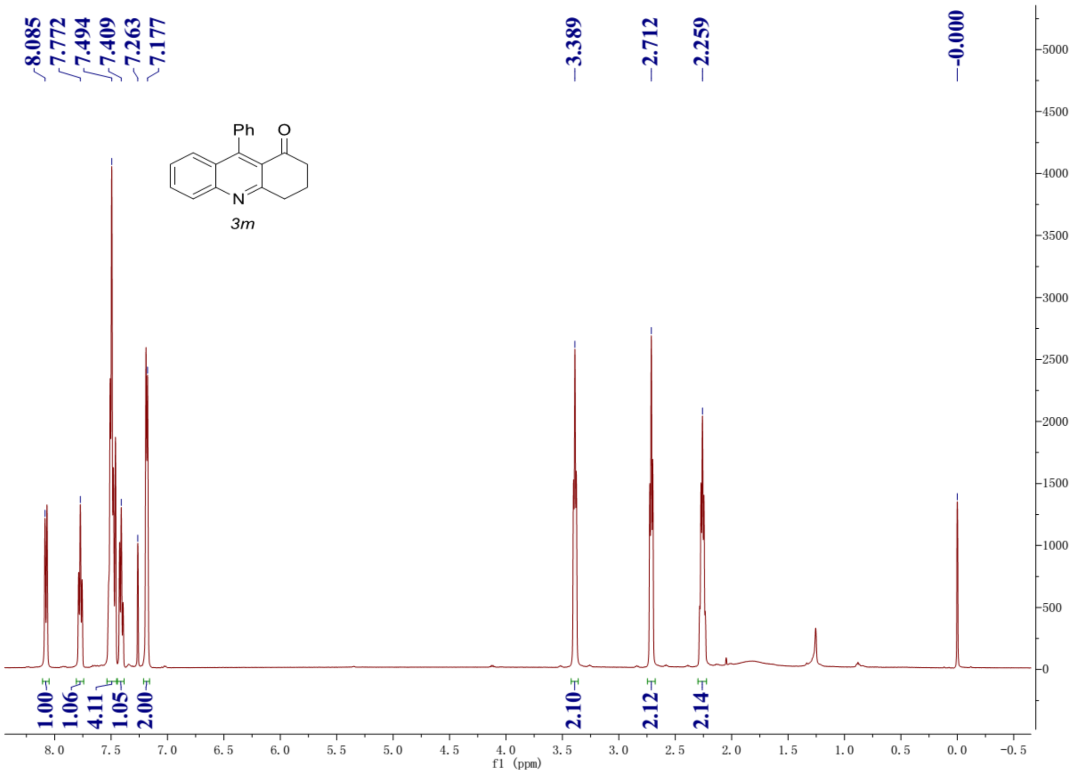


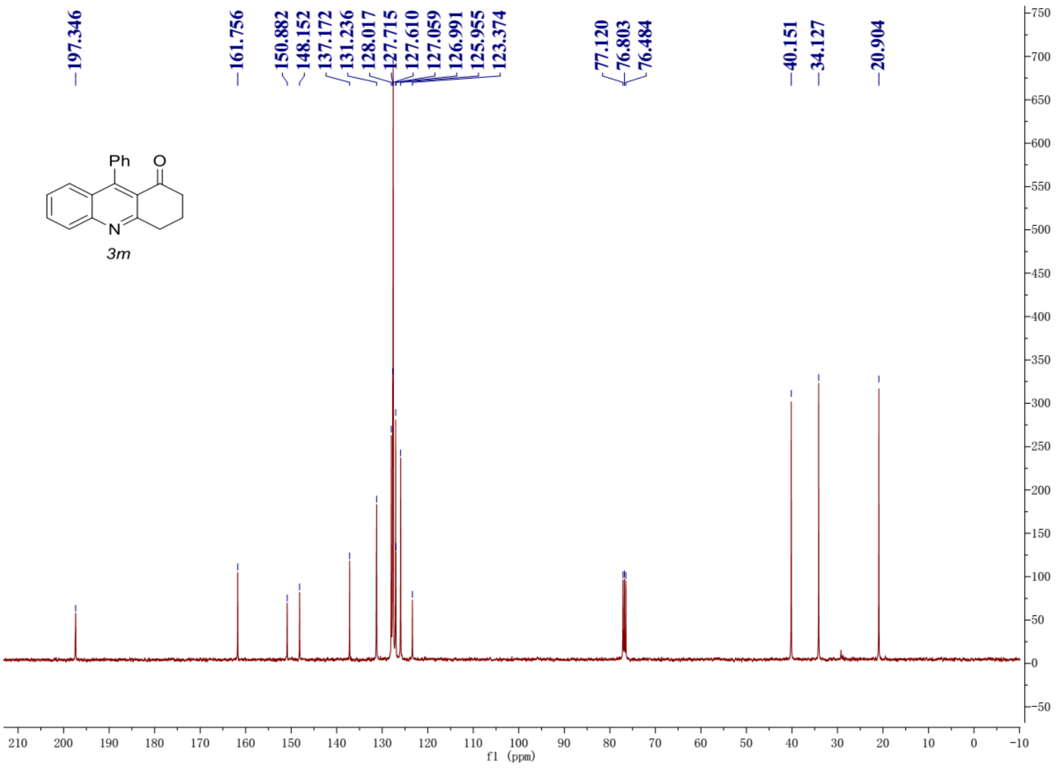

**1-(2,4-Diphenyl quinolin-3-yl)ethanone (3n)**

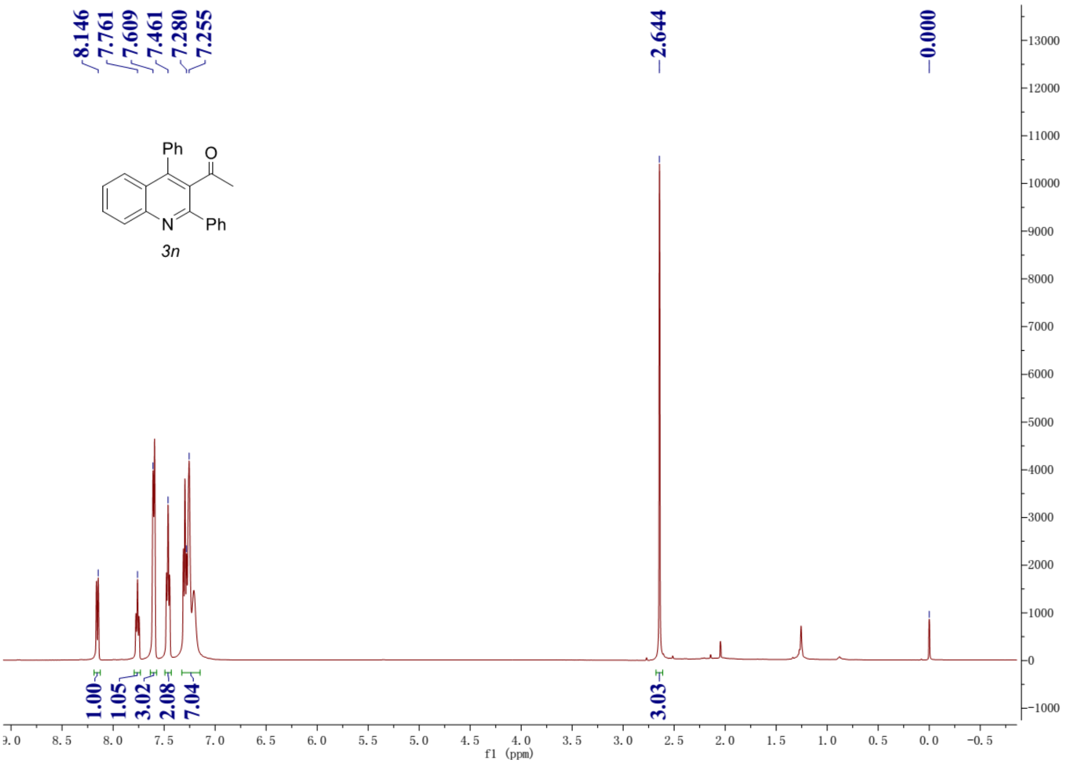


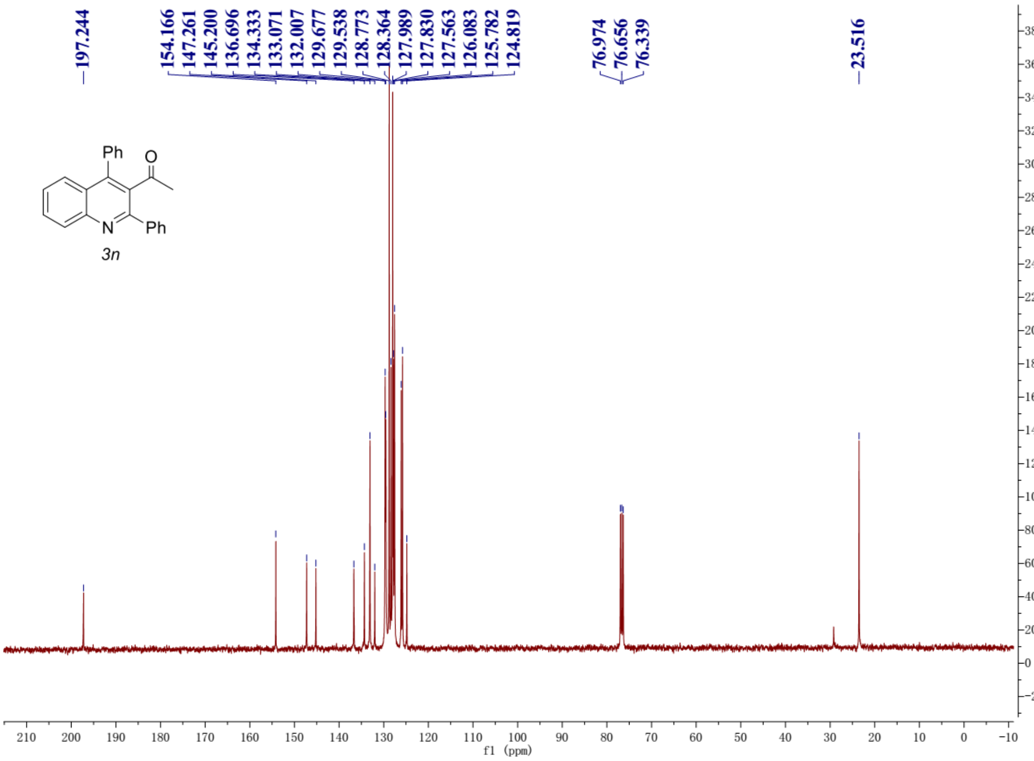

Supplement: Supplementary File 1 [file molecules-22-00762-s001.pdf]
